# Lignocellulosic Biomass for the Synthesis of Nanocellulose and Its Eco-Friendly Advanced Applications

**DOI:** 10.3389/fchem.2020.601256

**Published:** 2020-12-17

**Authors:** Guddu Kumar Gupta, Pratyoosh Shukla

**Affiliations:** ^1^Enzyme Technology and Protein Bioinformatics Laboratory, Department of Microbiology, Maharshi Dayanand University, Rohtak, India; ^2^School of Biotechnology, Institute of Science, Banaras Hindu University, Varanasi, India

**Keywords:** cellulose, flexible supercapacitor, energy harvesting device, nanocellulose, composite materials, water treatment, cellulose nano-fibrils (CNFs), cellulose nano-crystals (CNCs)

## Abstract

Nanocellulose is a unique and natural compound extracted from native cellulose using different extraction techniques. Nanocellulose is currently attracting attention due to its excellent properties such as special surface chemistry, exceptional physical and chemical strength, and rich hydroxyl groups for modification. In addition, its significant biological properties, like biodegradability, biocompatibility, and non-toxicity, accompanied by being environmentally friendly, are added advantages. The current review is focused on the lignocellulosic biomass processing methods for nanocellulose production and their usage for eco-friendly and environmental sustainability. We have also described insights into different techniques by which cellulosic materials can be changed into cellulose nanofibers (CNFs) and cellulose nanocrystals (CNCs). Lastly, we further discussed how nano-cellulosic materials are being used in a variety of industries such as the food sector, biomedical hygiene products, health care, water purification, and sensors. In the review, the unique uses of nanocelluloses in the production of nanocomposite materials, like flexible supercapacitor and polymer matrix, toward minimizing the utilization of global fossil energy and environmental pollution are envisaged. Finally, the significant application of nanomaterials in the areas of packaging industries, health and hygienic sector, cosmetics, and other important sectors are discussed. In the aspect of techno-economically feasibility, nano-cellulose-based materials may prove to be outstanding, environment friendly, and mitigate effluent load.

## Introduction

Environmental concerns are growing due to petroleum product usage. Hence, the production of superfine nanoscale efficient compounds from native cellulose is gathering more attention (Thakur and Voicu, 2016). Nanocellulose is a unique and promising natural compound derived from ordinary biomass. It is currently the most environmentally friendly compound that is techno-feasible and cost-effective, and also reduces effluent production (Kalia et al., 2011). Nanocellulose has retained significant attention due to its tremendous functionality i.e., greater surface chemistry, extraordinary biotic possessions, low toxicity, low in cost, lower density, and significant mechanical properties. The quick growth of green and nano-technological sustainability is related to the improvement of different resources and uses (Voicu et al., 2016). For 150 years, cellulose has been regarded as a renewable and biodegradable polymer, which has been used as an energy source and for various value-added products (Park et al., 2019). Previously, many researchers have been innovating bio-based compounds for the reduction of vestige fuel dependency (Mugaanire et al., 2019). Most of the cellulosic materials are utilized by many industries such as fabric, pulp mill, and reagent manufacturing sectors (Farooq et al., 2019). Recently, numerous investigators have been discovering efficient NC resources by using significant waste managing system (Lavoine et al., 2012).

In the global market, the business of fiber and its products is expected to achieve about USD 1.08 billion by 2020 and to continue to increase in value. Therefore, many resources are used for the production of cellulose and nanocellulose products. Among them, agro-waste residues are the most sustainable sources of cellulosic biomass, so various efforts have been prepared to mine cellulosic fiber from waste residues like fruits waste, marine biomass, and wheat straw (Yang et al., 2017). Nanocellulose has been used as filler in various applications and it enhances the physical strength of materials and barrier properties of carboxymethyl cellulose (CMC), chitosan, alginate, and biopolymeric films. Similarly, another study reported that nanocellulose is used in enzyme immobilization, automotive and electric appliances, and sensor devices (Farshchi et al., 2019). Various techniques, such as hydrolysis and physical and biological pretreatment methods, have been used for the production of cellulose fibers. These fibers convert/disintegrate into a crystalline, rod-shaped, and nano-sized units, such as much as micrometers, called Cellulose Nano-Crystal (CNC). However, the nano-sized compound can be produced using a microbial approach called bacterial nanocellulose (BNC) (Wijaya et al., 2019). Numerous studies have reported on Cellulose Nano-Fibril and CNC extraction from different types of sources such as bamboo, cotton, sugar beet, banana rachis, and agricultural waste, including hardwood and softwood (Haldar and Purkait, 2020a). Another report revealed that the powder form of Australian native “spinifex” grass *(Triodia pungens)* has been processed with an ultra-high pressure homogenization technique for the withdrawal of CNF (Amiralian et al., 2015). Habibi and coworkers in 2010 found that nanoparticles were extracted from the cotton, which has a dimension of 70–300 nm in length and 5–11 nm in width (Habibi et al., 2010). Similarly, Kang et al. reported that corncob wastage of ball milling was used as sources for CNF production through a step mechano-chemical esterification process (Kang et al., 2017). Additionally, Pandey and colleagues in 2015 revealed that the production of CNF through newspaper waste has a significant impact on the ecosystem and is economically important (Pandey et al., 2012). BNC has been extracted by using a bacterial submerged fermentation process. It can be used with several culture mediums, such as carbon source (glucose, agricultural waste, and fructose), nitrogen source (peptone yeast extract), and saccharified food waste. Typically, microorganisms like *Gluconacetobacter hansenii, G. xylinus*, and *Acetobactor* sp. are used for the fermentation process. Generally, after the bacterial treatment of cellulose, acid hydrolysis was employed to mine the BNC. However, expected and accurate proportions of BNC depends upon various optimized parameters (Haldar and Purkait, 2020b). Hence, the BNC production rate usually varies between 0.01 and 0.31 g L^−1^ h^−1^ (Reiniati et al., 2017). All three nano-celluloses (CNF, CNC, and BNC) have similarities, such as containing a native cellulose I crystal structure, having a great molecular weight, most polymerization properties, and having a significant ultrafine structure that is capable of holding them in water (Castro et al., 2011). Cellulose pretreatment can be done through several methods, like alkaline-acid pretreatment, enzymatic pretreatment, and ionic pretreatment, to remove the unwanted materials and enhance the nano-cellulosic properties. In the alkaline-acid treatment methods, the lignocellulosic compound is processed via alkali chemicals (Gupta et al., 2020a). These methods cause fibrillation and modified the surface properties of native fibers which way-out the hemicellulose, lignin, and waxes (Phanthong et al., 2018). After the chemical and biological purification of native lignin-cellulosic materials, the next methods are the transformation of refined cellulosic component nanosized particles (CNC, CNF, and BNC) through various methods, especially acid hydrolysis and physical treatments (Trache et al., 2016). Characteristics of various nanocellulose derived from lignocellulosic biomass and their applications have been described in Table 1. Various physical processes, like high-pressure homogenizers, microfluidization, cryo-crushing, and high-intensity ultrasonic treatments, are used to convert cellulose fibers into nanofiber materials (Ferrer et al., 2012). Nanocellulose materials are used in the production of bio-components, packaging component, polymer matrix, and antimicrobial barrier or coating materials in the food industry, as well as in the pharmaceutical sectors and most advanced 3D-structural technologies (like tissue or cell culturing process and diagnostic kits). These nanocellulose has been used for the production of nanocomposite materials, like flexible supercapacitor and polymer matrix, as they minimize the utilization of global fossil energy and environmental pollution (Abitbol et al., 2016). The current review focused on the lignocellulosic biomass processing methods for nanocellulose production and their eco-friendly and environmentally sustainable usage.

**Table 1:** Functional characteristics of various nanocelluloses derived from lignocellulosic biomass and their applications.

**Types of nanocellulose**	**Biomass sources**	**Production methods**	**Properties**	**Functional characteristics**	**Applications**	**References**
Cellulose nanofibers (CNFs)	Sugar beet, cotton, Soybean, jute, pea hull, hardwood; birch, poplar, eucalyptus	Chemical or enzymatic pretreatment and mechanical pretreatment followed by acid hydrolysis; TEMPO-mediated oxidation (Carboxylation);	Diameter: 5–60 nm Length: several μm; efficient roughness; high thermal stability;	Promote repulsion between cellulose fibers; Induce fiber separation; interruption in lignin composition;	Formation of nanocomposites; Strong additives; starch based plastic polymer; formation of solar cell; fuel cells	Puangsin et al., 2017; Peretz et al., 2019
Cellulose nanocrystals (CNCs)	Soft wood: Douglas fir, maize straw; rice straw; cotton; birch wood; waste pulp residues;	Acid hydrolysis followed by alkaline treatment/hydrolysis; Periodate-chlorite oxidation (Carboxylation); Sulfonation	From wood biomass Diameter: 5–70 nm Length: 100–250 nm; high in surface area; significant transparent;	Exploration and separation of cellulose nanofibers; Depolymerization of cellulose and hemicellulose;	Drugs delivery; blood vessels formation; food packaging; formation of PVA composite; production of nanogenerator;	Syafri et al., 2018; Farshchi et al., 2019
Bacterial nanocellulose (BNC)	wheat straw; pine; pulp and paper industry wastes; birch; rice husk; chemical medium: Simple sugars; disaccharides;	Bacterial culture; bio-fermentation process; Biological pretreatment; Through microbial enzymes: endoglucanase; cellobiases; lignin modifying enzymes; carboxymethyl cellulase	Diameter: 20–100 nm; Diameter: 5–70 nm Length: many μm; high thermal constancy; enhanced transparency; higher surface area.	Randomly cellulose hydrolysis; enhance cell wall softness; cleavage of lignin and hemicelluloses	Biomedical; biodegradable polymer; optical sectors; natural rubber; bio-composites; used in energy capacitor: supercapacitor electrodes.	Amiralian et al., 2015; Kang et al., 2017; Wijaya et al., 2019

## Nano-Cellulose Types and Properties

Bengt Rånby first described the nanocellulose material as cellulose bundles generating micelles in water solution in 1951 (Chen et al., 2018). Nanocellulose material can be extracted from woody biomass through conventional techniques, releasing the lignin and hemicellulose residues then converting the purified cellulose content into the nanoscale materials (Kargarzadeh et al., 2018). The term “nanocellulose” is roughly categorized into three different varieties: cellulose nanofibers (CNF), cellulose nanocrystals (CNC), and bacterial nanocellulose (BNC). These vary upon their sizes and functionality (Abitbol et al., 2016).

### Cellulose Nano-Fibrils (CNFs)

The extraction of cellulose nanofibrils (CNFs) from wood pulp biomass is often done using the TEMPO-mediated oxidation method. Its nanoscale is about 5–60 nm in diameter and many microns in length (Habibi et al., 2010). CNF consists of cellulosic domains i.e., crystalline and amorphous domains. Generally, several resources of native biomass, such as corn husk, rice straw, soft and hardwoods, and banana can be used for obtaining NCF (Farooq et al., 2020). In recent years, there has been only a low level of interest in the commercialization of CNF due to its high-cost production process. But recently, various chemical and enzymatic methods have been introduced for the production of CNF due to cost effective processes (Suopajärvi et al., 2017). The chemical methods, such as 2,2,6,6-tetramethylpiperidine-1-oxyl radical (TEMPO), periodate, and carboxymethylation, are the most used methods for the obtaining of NCF. The endoglucanase enzyme is used for enzymatic pretreatments of cellulosic content and the yielding of CNF materials (Barbash et al., 2017).

### Cellulose Nanocrystals (CNCs)

CNC is needle-shaped and highly crystalline and produced from cellulose pulp by acid hydrolysis process; through this process amorphous fibrils are dissolved to maintain the crystallinity. These materials contain cellulose moieties which are termed as cellulose nanowhiskers and microcrystalline (Clemons, 2016). The dimension of the yielded CNC is 5 nm in diameter and 20–100 nm in length. Similarly, another study reported that the average dimension obtained was 3–35 nm in diameter and 100–1000 nm in length (Nechyporchuk et al., 2016). Therefore, the NC structure varies depending on cellulosic biomass types and processing methods (Campano et al., 2016). According to Liu in 2014, CNC consists of highly strong material-like structures, such as Young's modulus, which is related to Kevlar material and used in the making of highly robust and tough materials (Liu et al., 2014). For commercialization purposes, many sectors started production and commercialization of CNC due to its tremendous properties. In India, the Indian Council of Agriculture Research produces about 10 kg/day of nanocellulose and commercialized their products in the market (Kumar et al., 2020).

### Bacterial Nano-Cellulose (BNC)

BNC are ribbon-shaped fibers with a usual size of 20–100 nm and are many micrometers in length (Jonoobi et al., 2015). BNC contains fibers with high crystallinity and high polymerization properties. BNC can be obtained through various bacterial strains like *Gluconacetobacter xylinus, Aerobacter, Escherichia, Sarcina, and Komagataeibacter*. Among these, *Gluconacetobacter xylinus* is the most widely used strain for the obtaining of BNC nanocellulose. BNC is secreted as an extracellular product from the bacterial fermentation process. Purification of BNC from cellulosic content occurs by the washing of bacterial cellulosic products using 1 M NaOH (Römling and Galperin, 2015). The elemental structure of BNC is quite similar to the high transparency level in comparison to plant-based biomass cellulose. The advantage of BNC is that it does not have unwanted polymers, unlike plant origin cellulose. BNC production from various microorganisms and their biomedical applications are given in Table 2. BNC materials have gathered the most interest in the biomedical field due to their tremendous activity in their nano-cellulosic materials (Jozala et al., 2016).

**Table 2:** BNC production from various microorganisms and their biomedical applications.

**Microorganism**	**Carbon source**	**Production method/time**	**Yield, g/L**	**Features**	**Applications**	**References**
*A. xylinum BRC 5*	Glucose	Static mode/50 h	15.30	Applicable for commercial scale, Absorbency and tolerance to fiber lift	For wound dressing composites, development of antimicrobial nanocomposite matrix	Kralisch et al., 2010
*A. xylinum subsp. sucrofermentans BPR2001*	Corn steep liquor-fructose (CSL-Fru) medium	Airlift reactor/67 h	3.8	Ability to stand with adverse conditions	For drug delivery applications, labeling as adhesive at low temperature	Huang et al., 2013
*A. xylinum BPR 2001*	Molasses	Static/agitated/72 h	7.82	Enhanced scale up process	Used for the sealing of surgical equipment	Kim et al., 2012
*A. subsp. Sucrofermentans BPR2001*	CSL-fru medium	Agitated/40 h	8.0	Wet strength	Used as packaging material of medical purposes	Silva et al., 2014
*A. xylinum NBRC 13693*	Pineapple medium and orange juice medium	Static/14 days	0.14	Biocompatibility	For nanocellulose based fabrication process such as facial masks and hydrogels for scaffolds	Lin et al., 2014
*G. xylinus ATCC 53524*	Sucrose	Static/96 h	3.83	Non-reactivity properties	Useful for the instruments packaging, making of nanocomposite materials for the drug delivering	Shao et al., 2016
*Acetobacter* sp. *V6*	Molasses and corn strip liquor	Agitated/168 h	3.12	biodegradability	For making of scaffolds, cell and tissue culturing, 3D printing of organs	Zhijiang et al., 2012
*G. persimmonis GH-2*	Glactose + Sucrose Watermelon + HS medium	Cell-free extract technology/14 days	7.67 and 5.93	Improved BNC production	Making of nanocomposite materials for the bearing of load during biomedical applications, nanocellulose immobilization for the affinity legends, and enzymes.	Baldikova et al., 2017
*G. intermedius SNT-1*	Molasses pretreated with heat	Static intermittent fed batch technology/10 days	12.6	Highly enhanced production as compared to standard static method,Can be applied for large scale production	Cell and tissue engineering application, especially bone tissue engineering	Dubey et al., 2018
*Acinetobacter* sp. *BAN1*	Pineapple waste medium (PIWAM)	Rotary disc reactor (RDR)/15 days	0.4-0.6	Mechanical strength	Enhancement of barrier properties, air permeability	Leitão et al., 2013
*K. rhaeticus iGEM*	Fermented tea	Static/10 days	NA	NA	Making of biosensors	Kim et al., 2013
*Beijerinckia fluminensis WAUPM53 and Gluconacetobacter xylinus 0416*	Sago by-product hydrolysate	Rotary disc reactor (RDR), Air lift reactor/14 days	0.4	Application orientation processibility, thermos stable	Oil absorbency, antimicrobial activity, diagnostic kits, and biosensors	Abbasi-Moayed et al., 2018

## Extraction of Nano-Cellulose From Lignocellulosic Biomass

**Figure 1:**
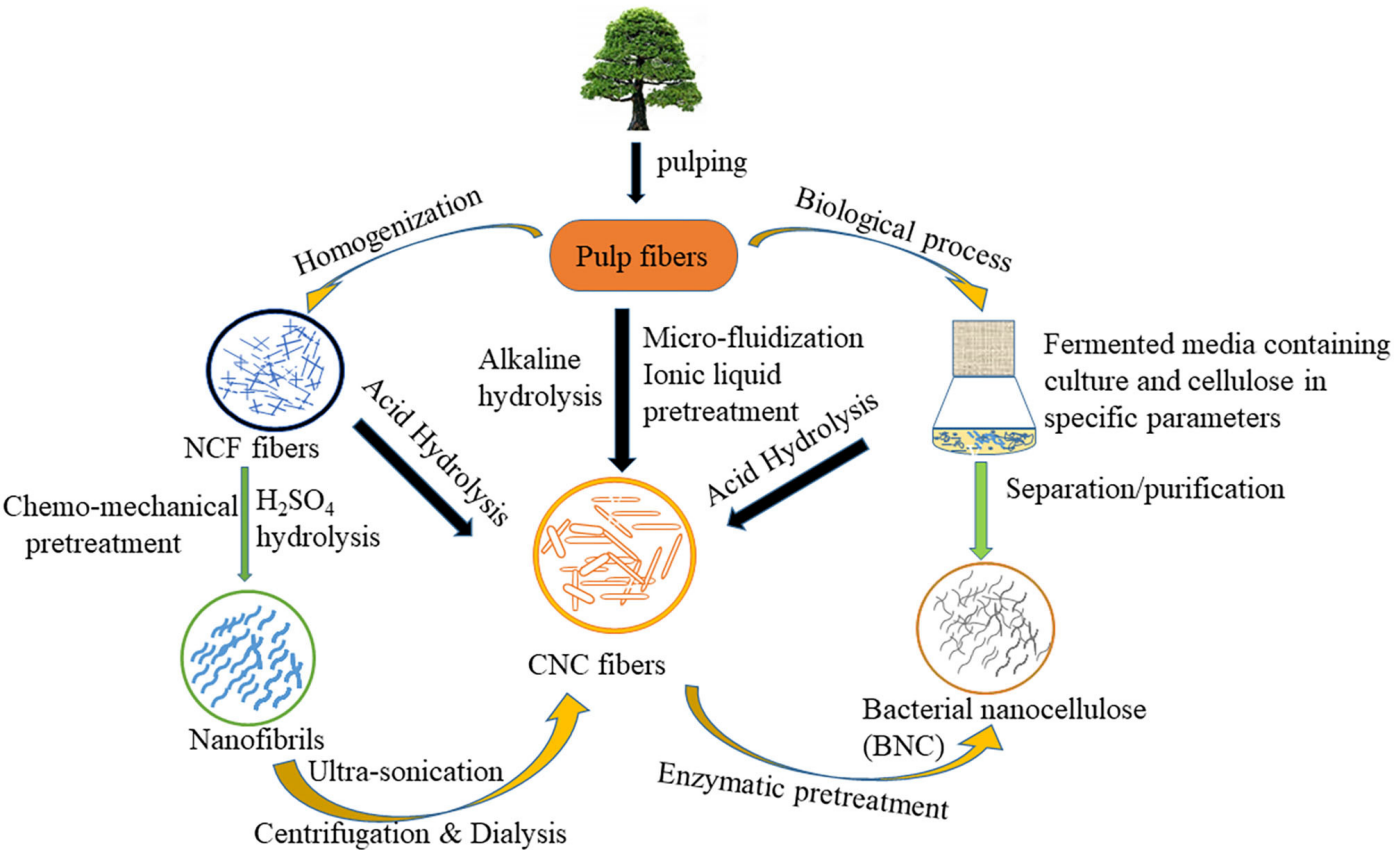
The schematic representation of nanocellulose production.

Nanocellulose can also be obtained from cellulose fibers through several treatment methods (e.g., traditional treatment and enzymatic treatment) of lignocellulosic biomass. The production of nanocellulose is attained by a two-step process. In the first step, the pretreatment process of native cellulose biomass is done which yields treated cellulose fibers. While in the second step, pretreated cellulose fibers are converted into nanocellulose using various routes e.g., high-pressure homogenization, micro fluidization, micro grinding, high-intensity ultra-sonication, electrospinning, and steam explosion. The schematic representation of nanocellulose production is given in Figure 1.

### Lignocellulosic Biomass Pretreatment

Agro-residue has gained attention as a resource for nanocellulose production. The uses of these waste products are not only based on their availability but also on the conversion of the valuable and high profitable product (nanocellulose) from the non-valuable waste (dos Santos et al., 2013). Moreover, the significant use of agro-waste is good for the ecosystem. As described above, lignocellulosic biomass is high in cellulose and undesired compounds, such as hemicellulose and lignin (Johar et al., 2012; Gupta et al., 2020b). The pretreatment process can be done before the extraction of nanocellulosic materials. To remove the ashes, waxes, and undesired polymers (i.e., hemicelluloses, lignin) and enhance the quality of cellulose, pretreatment process are needed (Gupta et al., 2020c). Also, the pretreatment process improved the fibrillation as well as the conversion of the cellulose fiber into micro/nanofibrils. However, the machine-driven process is a costly procedure due to its high energy consumption during the treatment process (Visanko et al., 2014). The pretreatment process also reduces the energy (from 20, 000 to 30, 000 kWh/ton to 1,000 kWh/ton) needed for the production of nanocellulose. There are several treatment processes, such as alkaline hydrolysis, acid hydrolysis, organosolvent treatment, ionic liquid treatment, and enzymatic treatment, that can be done for nanocellulose production. In the pulp and paper industry, acid-chlorite treatment or bleaching process is mostly used for biomass treatment (Hubbell and Ragauskas, 2010). Hubbell and coworker reported that it can remove most of the organic compounds through the combinatory effect of water and chlorite at optimized values, such as 70–80°C for 4–12 h. After that, the treated cellulose kept over a period minimizes the pH value till an ambient pH level is reached and then the cellulose is dried. Finally, the solid yields are termed as holocellulose (Li et al., 2012). The alkaline treatment, meanwhile, can be done for the removal of the amorphous polymer of an organic compound. These holocellulosic materials were treated with an alkaline chemical, i.e., sodium hydroxide, for a period of 1–5 h, treated with water until ambient pH level is reached, and dried (Abraham et al., 2013). Microbial processing is used for the alteration of cellulose biomass by degrading hemicellulose and lignin components. Various biocatalysts, such as endoglucanase, cellobiohydrolase, and hemicellulose, have been employed for the alteration of cellulose. In the biological method, a biocatalyst acts in either a restrictive hydrolysis process or a specific hydrolysis process of fiber components (Wahlström and Suurnäkki, 2015). The retention time for the enzymatic pretreatment needs to be much higher than in chemical pretreatment. Many researchers have reported that enzyme hydrolysis in combination with the homogenization of softwood cellulose resulted in nanocellulose with a greater aspect ratio that is less aggressive than the acidic treatment (Phanthong et al., 2018). Another study reported that microorganisms prepare cellulose naturally by using simple sugar molecules. Hence, the bioconversion of carbohydrates into cellulose is a multifaceted step and depends on several biocatalysts. Sometimes mutation can occur in the microbes and enhances the thickness of processing media, which is a demerit of this process (Chen et al., 2018). Researchers have studied and reported that a significant quality of cellulose is achieved on carbon sources i.e., glucose, mannitol, and fructose sugars (Islam et al., 2017). Besides traditional pretreatment, enzymes cellulose was found to be an eco-friendly process. Many researchers report that the synergistic effect of enzymes shows an excellent effect on the surface modification of cellulose biomass. Cellulases A and B, such as cellobiohydrolases, act on crystalline cellulose, whereas C- and D-type, i.e., endoglucanases, act on the amorphous cellulose. Cellobiohydrolase I and II are derived from *Trichoderma reesei*, which combine with cellulose-degrading catalyst and have high polymerization properties (Yarbrough et al., 2017). The BNC are also produced from the fruit and vegetable peels by the action of certain bacterial cells. In this process, vegetable peels are hydrolyzed by the acid hydrolysis and then by the bacterial cell for BNC production. Acid hydrolysis is performed at 10 mL/g liquid/solid ratio with 0.6 M H2SO4 for 2 h at 100°C using *K. hansenii* GA2016 microorganism. The highest BNC is produced from the kiwifruit and has the highest water holding capacity compared to other BNC (Güzel and Akpinar, 2020). Therefore, vegetable nanocellulose must first be chemically isolated using sequential reagent processing with alkaline solutions at various doses. After the chemical treatment, a physical process must be carried out which would breakdown the cellulose and provide 5–20nm sized nanocellulose (Klemm et al., 2011). The most preferred mechanical process are high pressure homogenization, microfluidizer, waring blender, and grinder (Uetani and Yano, 2011).

### Nano-Cellulose Extraction

Various technologies have been discovered for the isolation of nanocellulose from pretreated cellulose. In these scenarios, the isolation methods are mainly focused on three categories: mechanical extraction, enzymatic hydrolysis, and acid hydrolysis (Anwar et al., 2014). Among these, acid hydrolysis is most widely used for the extraction of nanocellulose from biomass fibers. It is easily hydrolyzed into the disordered regions of cellulose. In this process, sulfuric acid is widely employed for the acid hydrolysis. The mode of action of acid hydrolysis is dispersal of the nanocellulose via sulfate ions during the esterification process (Morais et al., 2013). A major demerit of acid hydrolysis is the generation of water waste during the wash away process of pretreated nanocellulose. TEMPO oxidized cellulose nanofibers consist even with a high aspect ratio and have a broad range of applications, including a gas-barrier film for packaging and nanofiber filling for composite materials (Fukuzumi et al., 2009). Biological hydrolysis can be done using cellulose-digesting enzymes. Generally, a high reaction time is needed during the enzymatic hydrolysis process. However, to overcome this problem a combination of enzymatic and other methods are employed for the digestion and extraction of nanocellulose (Khalil et al., 2014). Moniruzzaman and Ono in 2013 found that the cellulose separated from wood biomass with ionic pretreatment, which enhanced the available exterior zone and then causes bio-catalytic activity to take place. The NC produced by this method has significant crystallinity and high stability as compared to ordinary fibers (Moniruzzaman and Ono, 2013). Many researchers in the 21^st^ century are focused on the factors' optimization for BNC extraction to obtain a significant quality of BNC using traditional media, consisting of sugars at static or agitated operations (Sharma and Bhardwaj, 2019). The novel substrates (often rich in sugars) like sugarcane scum, pineapple waste, and coconut water instead of traditional sugar source can be used for BNC production. The microorganisms, especially bacterial genera like *Acetobacter, Bacillus, Sarcina, Rhizobium, Enterobacter*, and *Klebsiella*, are recognized for BNC production. However, gram-negative bacteria such as *Gluconacetobacter xylinus*, formerly known as *Acetobacter xylinum*, are the most common bacteria reported for the BNC production (Siró and Plackett, 2010). The extracted cellulose from the *Gluconacetobacter xylinus* has similar characteristic features to plant cellulose, which is observed using X-ray diffraction technique. The nanocellulose produced from the *Gluconacetobacter xylinus* have β-1,4-glucan chains and a twisted and ribbon-like structure in the environment. The purity of formed cellulose can be estimated using the cellulose solubility test method. In this method, cupriethylenediamine and sodium hydroxide are used, and complete solubility of cellulose indicates purity level. Therefore, batch and fed-batch methods are used for the static production of BNC through bioreactors. The BNC extraction is directly subjected by its extraction techniques and factors involved during the production process (Gama et al., 2016). BNC production at a commercial scale might be achieved through advanced bioreactor methods. According to Kralisch et al. in 2010, BNC foils were produced with desirable lengths and thicknesses using a horizontal lift reactor at static condition (Kralisch et al., 2010). Recently, Dudey and co-workers used static intermittent fed batch technology for BNC production and reported that they obtained 38 g/l BNC using *Komagataeibacter europaeus* SGP37 strain in sweet lime pulp waste medium (Dubey et al., 2018). Another type of bioreactor, rotatory biofilm contactor (RBC), was used by Kim et al. in 2007 who reported 5.52 g/l BNC production while using *Gluconacetobacter* sp. (Kim et al., 2007). Most researchers used substrate-based strategies for BNC production using several microorganisms. However, Son and colleagues in 2001 revealed that, using *Acetobactor* sp. A9, BNC production improved 4-fold while using ethanol addition as supplemented agents (Son et al., 2001). Kim et al. concluded that the addition of agar in the production media increased the BNC productivity (Kim et al., 2012).

## Advanced Application of Nano-Cellulose

**Figure 2:**
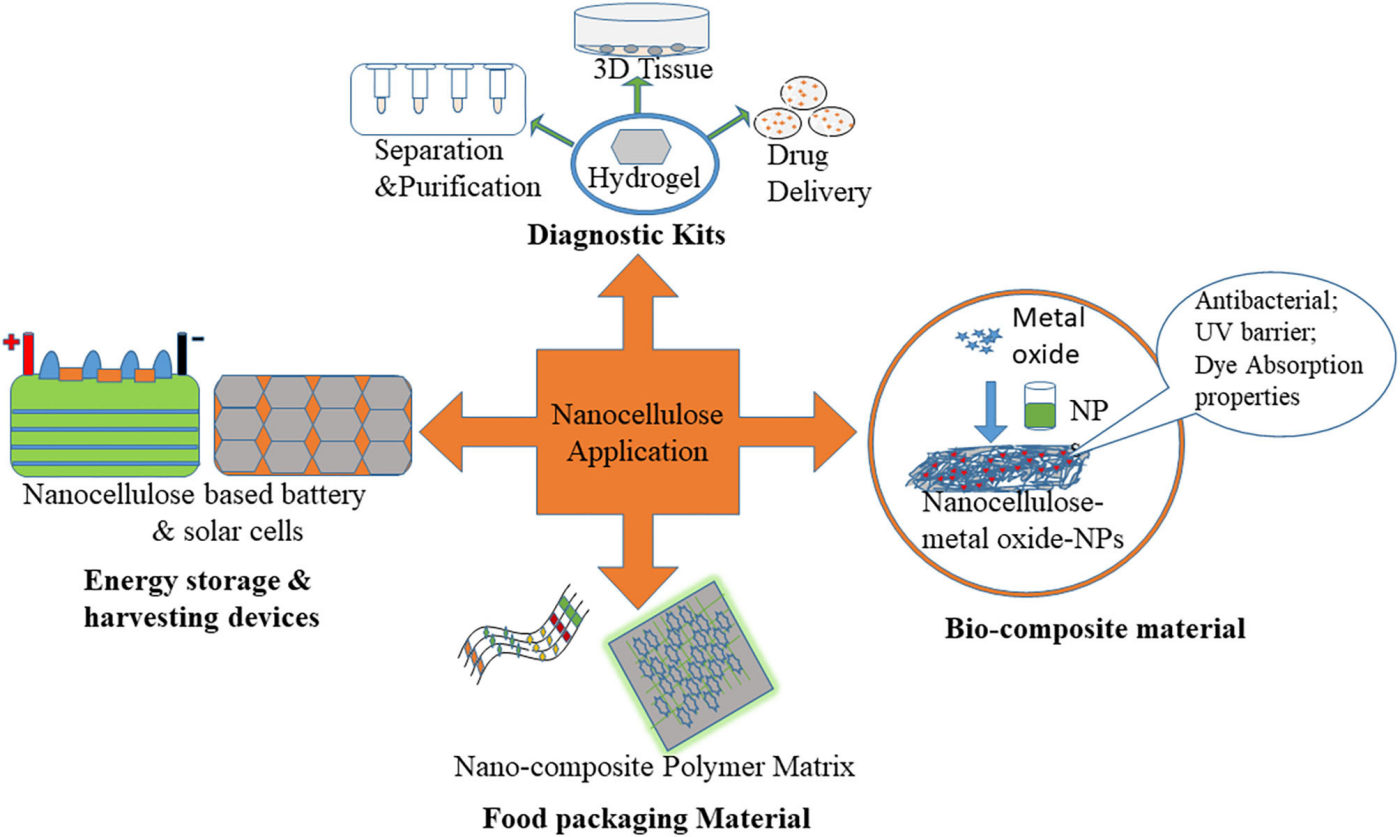
An overview of various applications of extracted nanocellulose.

Due to outstanding functionality and being ecologically friendly, nanocellulose is used in many fields such as nanocomposite materials, surface modified materials, and transparent paper with distinct properties. We discussed how nanocellulosic materials are utilized in a variety of sectors such as the nourishment sector, biomedical products, health care, water purification, and sensors. An overview of various applications of extracted nanocellulose is depicted in Figure 2. These nanocellulose have been used for the production of nanocomposite materials like flexible supercapacitor and polymer matrix; they also minimize the utilization of global fossil energy and environmental pollution.

### Energy Conversion and Harvesting Devices

Nanocellulose paper has tremendous physical capacities (transparent, optically clear, and foldable) and is used in electronic appliances, energy capacitors, harvesting devices, and as flexible composite materials (Salas et al., 2014). Nogi et al. in 2009 stated that transparent nanocellulose paper was fabricated from timber powder, and reveal its optically active, excellent physical strength and high modulus (13GPa) (Nogi et al., 2009). Weng and coworkers used simple fabricated graphene-cellulose paper (GCP) membranes as electrodes in the flexible supercapacitors. They found it has the greatest stability of electrical conductivity with only a 6% reduction after being bent 1,000 times (Weng et al., 2011). Similarly, Cui and team reported that the commercial paper sheets utilized in the fabrication of supercapacitors and lithium-ion batteries were coated with carbon nanotube (CNT) ink with the help of sodium dodecylbenzene sulfonate (SDBS) as a surfactant (Jose et al., 2019). Cui's group reported that CNF-based organic solar cells have a power conversion efficiency (PCE) of 0.4%. However, the highest observed PCE of CNF-based solar film was about 4% (Hu et al., 2013).

### Biomedical Application

BNC is considered to be the most unique and cost-efficient natural nanomaterial for health sectors. It has extraordinary applications, such as scaffolds fabrication, wound-dressing, drug delivery applications, diagnostic and biosensors, and the most advanced tissue and cell engineering techniques. There are many biomedical applications of nanocellulose-based materials, most of which are highlighted here.

#### Antimicrobial Wound Dressing Agent

Nanocellulose that are non-hazardous, renewable, and have significant compatibility excellent durability are often used in pharmaceutical sectors. Hakkarainen and coworker in 2016 extracted nano fibrillated cellulose from bleached birch pulp and used it as wound-dressing and also reported that it has an ecofriendly use with graft systems (Hakkarainen et al., 2016). These dressing materials can be fitted with a wound and simply removed by a patient once the wound has healed (Abitbol et al., 2016). Along with this, cellulose modification is enhanced by cell attachment. For this purpose, in various ways, such as the plasma treatment process, the protein coating process can be done. There are many nanocellulose-based antimicrobial composites, such as silver nanoparticles and lysozyme adopted with special effects. According to Fontana et al. in 1990, BNC-based “BioFill®” material is reported as the most anticipated wound dressing biomaterial in the biomedical field (Fontana et al., 1990). The BNC obtained dressing agents, like Bioprocess, XCell, and Gengiflex®, are commercially found for the treatment of periodontal disease reconstruction applications (Sharma and Bhardwaj, 2019). Similarly, BWD, XCell®, and Xylos formed from biocellulose can be applied for chronic venous leg ulcers (Alvarez et al., 2004).

#### Drug Delivery System

Nanocellulose is also applied in medical fields as bold vessel replacements, implants of soft tissues, and drug delivery to targeted cells (Jorfi and Foster, 2015). Nowadays, the use of nanocellulose in the drug delivery system has been adopted to monitor the systems. The hydrogel-based nanocellulose has been used as a drug carrier molecule, as it produced the stability of drugs (Gupta et al., 2019). Most of the researchers reported that the BNC-based carrier molecules can be used for the delivery of berberine hydrochloride and berberine sulfate drug delivery system (Huang et al., 2013). Similarly, tetracycline hydrochloride was blended with BNC composite material to be employed for drug delivery in skin dressing applications (Shao et al., 2016). However, the BNC- caffeine material was exploited for both solution and forms of the topical drug delivery application. Similar experiments on BNC-glycerin were conducted by many researchers who concluded the same topical drug delivery application (Silva et al., 2014). But an adverse effect (skin irritating problem) of BNC was found in human beings. Recently, BNC has acted as a biocarrier of 1,3-Dihydroxy-2-Propanone (DHA) for curing vitiligo. The BNC is the most promising biomaterial for the drug delivery system due to its ultrafine 3D nanostructure. Various studies reported that BNC can be responsible for delivering drugs, antibodies, and enzymes to their targeted sites (Abeer et al., 2014).

#### Cell and Tissue Culturing Techniques

BNC is noted as a unique scaffold component due to its tremendous characteristics. It can be divided into three scaffolds: BNC membrane scaffolds, BNC bio-composite matrix, and BNC pellicle scaffolds (Lin et al., 2014). BNC-based novel small caliber vascular prosthesis was invented by many researchers (Leitão et al., 2013). The BNC-gelatin materials can be used for the attachment of fibroblast cells. Using polymeric P(3HB-co-4HB) as a matrix, BNC bio-composite scaffold was formed with the help of the freeze-drying method and used for Chinese hamster lung fibroblast cells adhesion (Zhijiang et al., 2012). Another study reported that, BNC has significant efficiency in bone tissue engineering technology. The nanocellulose-based hydrogels have been effective with mesenchymal stem cells (MSC) for 3D culturing. The maintained rates of MSCs cell viability on the cellulose matrix are nearly 90% and have high distribution properties across the gel (Azoidis et al., 2017). Similarly, engineered tissues also required an ECM system for 3D bio-printing. Recently, hydrogels derived from NC have been discovered and employed as scaffolds in the 3D biological system. The BNC tube fabrication was achieved through the use of a polydimethylsiloxane template, which has been applied as artificial blood tubes with improved mechanical strength, huge temperature stability, and compliance concerning *in vivo* surroundings (Zang et al., 2015).

#### Diagnostic and Bio-Sensing Devices

Diagnostic kits and biosensors are employed for the detection and diagnosis of diseases in clinical experiments. Nowadays, various biosensors have been developed for different applications in biomedical fields. Generally, receptors, antibodies, antigens, and enzymes have been used as active factors for clinical evaluation (Sharma and Bhardwaj, 2019). However, using thiosalicylic acid and 2,2-dithiodipyridine as analytes, BNC-silver nanocomposite matrix has been applied for the biological analysis of surface enhanced Raman scattering (SERS) lamellas (Marques et al., 2008). Similarlythe advanced BNC-Au nanomaterials have been used for the detection of glucose compounds in the human blood serum. Currently, high performance artificial tongues were developed with the help of BNC nano-paper as source (Abbasi-Moayed et al., 2018). These nanopaper-based tongues have advanced biochemical disparity and can be used for the optical sensor array application. Thus, BNC-filtered carbon nanotube composites were developed for the immobilization of electrons within glucose and glucose oxidase (Kim et al., 2013). Additionally, BNC produced from *Komagataeibacter sucrofermentans* was altered with perchloric acid stabilized magnetic fluid and was applied as a transporter for the immobilization of cells, ligands, and enzymes in biomedical fields (Baldikova et al., 2017).

### Nanocomposite Materials

Nanocomposite materials obtained from nanocellulose consist of a few superior assets, such as high durability and wide-range stability while being low in weight (Abitbol et al., 2016). These nanocomposite materials have been applied in many fields, such as the blades of windmills, lightweight armor, and flexible energy capacitor. Robles et al. in 2016, used nanocomposite materials obtained from PLA and nanocellulose, and concluded that nanocellulose improved the contact between matrix fillers with thermal capability and enhanced crystallinity (Robles et al., 2015). Similarly, Ardanuy et al. reported on a sisal fiber cement composite produced by the CNF and sisal fibers and found 40% enhanced flexural strength as compared to ordinary cement composite (Ardanuy et al., 2012). The introduction of filler in the polymeric material improved the physical and thermal properties of the composite material, as compared to polymeric material alone (Xu et al., 2016).

### Sorbent Matrix for Liquid Materials

Various studies have been performed in the last few decades on the uses of nanocellulose in the oil drilling industry. The researchers Saboori et al. and Fereydouni et al. in 2012 found that the nano-scaled carboxymethyl cellulose (CMC) and polyanionic cellulose when used as fluid loss additives (Fereydouni et al., 2012; Saboori et al., 2012). The amphiphobic nanocellulose modified paper was fabricated by Phanthong et al. (2016). For this process, filter paper was coated by nanocellulose and then reacted with trichloro (1H,1H,2H,2H-tridecafluoro-noctyl) silane for deposition of vapor. This paper acted as a repellent to polar as well as non-polar liquids in the ecosystem due to its superhydrophobicity and oleophobicity properties. The biodegradable aerogels are produced from the nanocellulosic materials, consisting of high porosity and negativity charge with excellent adsorption capacity. Similarly, Li and coworkers studied the use of CNC as a rheology convertor. They reported that mud produced from nanocellulose and bentonite water have excellent rheological activity, high-temperature constancy, and low fluid loss (Li et al., 2015). Recently, nano-scale additives used in cement slurry formulation are considered for the enhancement of cement properties. With a high surface area, chemical reactions can be efficiently enhanced and significantly promote the degree of hydration (Ramasamy and Amanullah, 2020).

### Nanocellulose in Food Industries

The high surface area, aspect ratio, rheological behavior, and non-cytotoxic and non-genotoxic properties of vegetable nanocellulose mean it is easy to use in the food sector. This nanocellulose has been widely used in three main applications: as a food stabilizing agent, as a vital food constituent, and as food packaging. It is used as a food packaging application worldwide (Serpa et al., 2016). Nanocellulose is used as a food stabilizing agent for a variety of foods like soy sauce and soup, retort food, dough-based food, and whipping cream by several Japanese companies, such as Daicel, Procter & Gamble, and Asahi Foods Co., Ltd (Winuprasith and Suphantharika, 2015). A recent patent was filed by Yano and co-workers in 2014 who reported that nanocellulose used as a food stabilizer means frozen dessert can retain their shape. Another study revealed that nanocellulose used as dietary fibers have a range of beneficial roles. It can be used in the reduction of risk of chronic diseases like diabetes and cardiacarrest. Additionally, it improved physiological effects, including laxation and blood sugar level (Andrade et al., 2015). Nanocellulose-based film has extraordinary properties of air and oxygen barriers, which protect the food from undesirable entities. The characteristics of nano-based films are similar to natural poly bags. The excellent oxygen tolerance capability of nano-film arises due to the high crystallinity of nanocellulose (Svagan et al., 2016).

## Commercial Aspects

Numerous studies on the estimating and fabrication of nanocellulose have been reported by several organizations and industries (Mhd Haniffa et al., 2016). The US Forest Service reported that nanocellulose could add $600billion to the US economy by 2020. A similar study reported that ~1 × 10^12^ tons of cellulose and its derivatives were produced annually, which accounts for 1.8 × 10^9^ billion USD business by 2020 (Thomas et al., 2020). According to Gama et al. (2016), in the Philippines and Indonesia about 1,500 tons of BNC is produced and commercialized from the nata de coco farms (Gama et al., 2016). Various factors were involved to decrease the BNC production costs, like use of alternative sources, upgrading the scale up process, and the designing of bioreactors. However, the production cost remains a challenge. While in NFC, similar costs are absorbed on the pre-treatment process. The approximate cost of NFC processing from wood pulp is about 0.4 $/kg when using enzymatic pre-treatment approaches and is also reported as cheapest among the other process (Ålander et al., 2017). NFC produced by TEMPO-modified process has been reported as reducing the final processing cost. It has higher greenhouse gas emission during NFC production in comparison with other industries (Moon et al., 2017). Similarly, NCC shares the same expenditures with commercialization production via the sulphuric acid hydrolysis technique. During the NCC processing, a by-product is generated and can be used for bioenergy generation. However, the extra cost and energy are needed for the production of biofuel. Currently, various reports revealed that the cost-effective processes for NCC production are subcritical water processing, hot water pre-extraction, and oxidation techniques. Hence, the commercial price was estimated to be lowered by 18 $/kg for NCC production via using H_2_SO_4_ hydrolysis (Bian et al., 2017). According to NanoCrystalline Cellulose, 2019 report, Celluforce is reported as the highest NCC producing plants among commercial aspects, which has a production capacity of ~300 tons annually (NanoCrystalline Cellulose, 2019). Thus, it is challenging to analyze and predict the final cost of nanocellulose but it is estimated at between 7-12 USD/kg of final product (Thomas et al., 2020).

## Challenges and Future Perspectives

The presence of undesired organic materials, like lignin, hemicellulose, and its derivatives in the lignocellulosic biomass, present a challenge in the production of cellulose. The pre-treatment process is necessary for the removal of all these undesired organic compounds. Due to the time taken, toxic chemicals consumed, and discharged wastewater, the pretreatment process is a complex process. However, the conventional technologies have drawbacks like production of huge wastewater effluent, high energy demand, and long retention time for the production of nanocellulose using the acid hydrolysis process. Hence, enzymatic treatment has the efficient capability to extract out nanocellulose from the cellulosic biomass. Due to the current deand for flexible and sustainable energy devices, there has been a focus on ecological considerations in cellulose production. Certainly, challenges do exist and efficient research outputs are needed to overcome these issues to create innovative materials for sustainable applications. Consequently, more research and environmentally friendly processes are needed for the manufacture and alteration of nanocellulose as well as reaction activity. We presume that research on nanocellulose-based bio-decomposable polymers and porous nanocomposites will continue to grow. We must continue with the development of novel production processes of nanocellulose and its products. Furthermore, nanocellulose products are successfully and significantly expand them-self in upcoming nano-generation.

## Conclusions

The main focus of this review was to consider the extraction and application processes of NC from biomass. Nanocellulose is a promising material due to its distinctive functionalities i.e., high strength, low density, transparent, large aspect ratio, etc. The biomass pretreatment is a vital step to remove undesired materials. The BNC is the most preferred choice for drug delivery and pharmaceutical applications. However, for rheological activity, composite and strength additive NFCs would be the superior choice. In the case of reinforcing agents in biomedical applications, NCC is the significant choice. The NC-based conductive materials have been widely applied in the area of energy devices, such as in supercapacitors, solar batteries, and solar plants. Thus, the various materials obtained from CNF, CNC, and BNC would draw interest from various sectors e.g., pharmaceutical, food processing, and antimicrobial activity. Overall, nanocelluloses have multiple uses that can resolve several challenges of modern society and also play a vital role in the future of material science.

## Author Contributions

GG wrote the first draft of the manuscript. The final draft was read and edited by PS. Both authors listed have made a substantial, direct and intellectual contribution to the work, and approved it for publication.

## Conflict of Interest

The authors declare that the research was conducted in the absence of any commercial or financial relationships that could be construed as a potential conflict of interest.
